# Excess Weight and Body Fat Percentage Associated with Waist Circumference as a Cardiometabolic Risk Factor in University Students

**DOI:** 10.1155/2022/1310030

**Published:** 2022-01-05

**Authors:** Roberto E. Nuñez-Leyva, Tabita E. Lozano-López, Yaquelin E. Calizaya-Milla, Sergio E. Calizaya-Milla, Jacksaint Saintila

**Affiliations:** ^1^Escuela de Posgrado, Unidad de Posgrado Salud Pública, Universidad Peruana Unión, Lima, Peru; ^2^Grupo de Investigación en Nutrición y Estilos de Vida, Escuela de Nutrición Humana, Universidad Peruana Unión, Lima, Peru

## Abstract

**Background:**

Obesity is one of the most important public health problems for university students. The objective of the study was to evaluate the association between body mass index (BMI) and body fat percentage (%BF) with waist circumference (WC) as a cardiometabolic risk factor (CMR) among university students.

**Methods:**

A cross-sectional study was carried out in 2,048 students from a private university located in Lima, Peru. Anthropometric data (weight, height, %BF, and WC) were collected. Chi-square test was used. Association analysis was performed using multiple logistic regression.

**Results:**

The findings indicated that 36.9% and 61.1% of men were overweight and had higher %BF, respectively, compared to women. Women (OR, 0.22; 95% CI, 0.17, 0.29), Peruvian students (OR, 0.59; 95% CI, 0.39, 0.90), and students enrolled in the faculty of health sciences (OR = 0.76; 95% CI, 0.62, 0.94) are less likely to manifest CMR. Also, excess body weight (OR, 17.28; 95% CI, 13.21, 22.59) and a high %BF (OR, 4.55; 95% CI, 3.55, 5.84) were strongly associated with CMR.

**Conclusion:**

CMRs are a public health problem among university students. Therefore, it is important to carry out healthy lifestyle programs to promote better control and prevention, particularly among male students and those who have excess weight and body fat.

## 1. Introduction

Obesity constitutes one of the most important public health problems in the general population, particularly among university students [1]. Apart from the dire consequences on the economy of the countries [2], excess body weight represents a risk factor for cardiometabolic diseases such as type 2 diabetes and cardiovascular diseases [3]. These pathological conditions lead to deterioration in the quality of life and an increased risk of mortality. In Peru, according to the Instituto Nacional de Salud, 42.4% of young people represented by the university population have obesity [4]. Moreover, cardiometabolic pathologies represent the main cause of death in Peru [5]. In 2016, it was estimated that 16% of the Peruvian population over 20 years of age suffered from cardiovascular diseases and more than 2,000 deaths were due to some type of heart failure [6].

Cardiometabolic diseases, excess body weight, and elevated %BF in college students are the result of inadequate nutrition characterized by excessive consumption of foods of high caloric density, deficient in micronutrients and essential bioactive elements, and lack of regular physical activity [6, 7]. The World Health Organization (WHO) emphasizes that the risk of morbidity and mortality is the result of a series of risky eating behaviors including unhealthy eating practices that begin during the teenage and college stages [8]. For example, in Peru, only 11.3% of the population over 15 years old consumes five servings of fruits and/or vegetables a day, as recommended by the WHO [9]. Likewise, it is reported that no region of the country approaches this average consumption, which is ideal for preventing the aforementioned pathological conditions [9]. On the contrary, both in this age group and in university students, what is evidenced is a high consumption of added sugars, processed meats, and saturated fats higher than the daily intake recommended by the WHO [10]. Adequate consumption of minimally processed plant foods plays an important role in the prevention of various chronic conditions [11].

The university environment represents a real health challenge for students because they can have greater access to foods that are high in saturated fat, sodium, and free sugars; consequently, it leads to an increased risk of excess body weight and cardiometabolic diseases [12]. In addition, many of the lifestyle habits that were formed in this period can persist over time, affecting health in adulthood [13]. University students are a vulnerable group for the development of obesity and cardiometabolic diseases [14]. Some studies have shown that college students are at greater risk of gaining weight than those who do not attend college. For example, findings from a study conducted on Norwegian university students reported that the prevalence of overweight/obesity increased substantially among university students, from 29% in 2010 to 36.4% in 2018 [15]. Similarly, Albaker et al. [16] in a recent study, they highlighted a high prevalence of adiposity and CMR among university students.

Choosing healthy foods is increasingly challenging for young adults, especially after the transition to adolescence, a time characterized by independence [17, 18]. On the one hand, during this period, other frequent behaviors related to weight gain are observed, such as excessive alcohol consumption, skipping breakfast, high consumption of fried foods, and increased sedentary lifestyle. On the other hand, the transition from high school to university life involves substantial changes in the lives of young adults [18]. Therefore, there is a clear need to explore, identify, and evaluate these anthropometric parameters (weight, height, BMI, %BF, and WC) to better understand their impact and contribute to cardiometabolic pathologies to improve the general health and well-being of college students [19]. In addition, expanding our knowledge of these issues in this population is of great importance because excess weight and body fat can lead to serious health problems. Consequently, the purpose of this study was to evaluate BMI, %BF, and its association with WC as a CMR factor among university students.

## 2. Materials and Methods

### 2.1. Design and Participants

**Figure 1:**
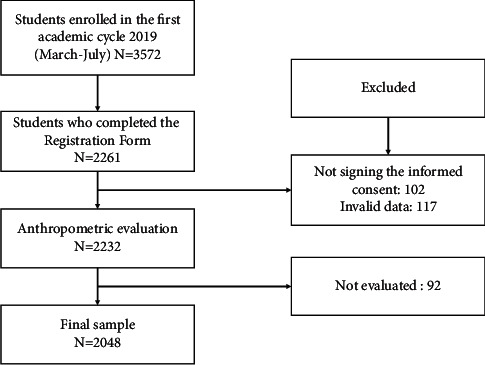
Study design.

A cross-sectional study was conducted in a private university located in the eastern zone of the city of Lima (capital of Peru) at the beginning of the 2019 academic year. Participants ranged in age from 20 to 29 years. Data collection began on January 27 and ended on March 18 of the same year; one week after the official start of classes was announced by the university authorities. All university entrants (*n* = 3,572) were selected through the nonprobability convenience sampling technique to participate in the study. However, *n* = 2,261 decided to participate in the study. Participation in this study was completely voluntary. Students who underwent medical treatment were not chosen to participate in the study. Additionally, those who did not sign the informed consent form (*n* = 102), who did not register valid sociodemographic data (*n* = 117), who were not evaluated anthropometrically (*n* = 23), and those who were under 18 years of age (*n* = 69) were excluded, and finally, the final sample was *n* = 2,048 students (Figure 1).

### 2.2. Ethical Aspects

All students were initially informed of the objectives of the study and the purpose of data collection. They also signed a written informed consent form giving their authorization to participate in the study. The study was developed according to the international standards proposed by the Declaration of Helsinki. In addition, it received the approval of the Research Ethics Committee and registered at the following number: 016-2019/UPeU/FCS.

### 2.3. Sociodemographic and Anthropometric Data

We have used a sociodemographic and anthropometric data registration form to collect specific information on age, sex, place of origin, and marital status. Moreover, the educational profile that includes study faculty (theology, humanities, health sciences, business sciences, engineering, and architecture) was considered. Finally, information was measured on the weight, height, BMI, %BF, and WC of the students.

### 2.4. Anthropometric Measurements

#### 2.4.1. Body Mass Index (BMI)

Data on weight and height were collected; for this, the participants were told that they should be without shoes to favor a better measurement. Both anthropometric indicators were measured using a calibrated SECA 700 brand mechanical column scale, capacity: 220 kg, and measuring range: 60 to 200 cm (SECA®, Hamburg, Germany). The BMI was calculated according to the parameters established by the WHO and classified as follows: underweight, BMI <18.5 kg/m^2^; normal weight, BMI from 18.5 to 24.9 kg/m^2^; overweight, BMI from 25.0 to 29.9 kg/m^2^; obesity, BMI ≥30 kg/m^2^.

#### 2.4.2. Cardiometabolic Risk (CMR)

On the other hand, CMR was determined by measuring WC using a metal steel self-retracting tape measured by Cescorf (Cescorf Equipamentos Para Esporte Ltda-Epp, Brazil). CMR was considered for a waist circumference ≥94 cm in men and ≥80 cm in women. These parameters were established in the Peruvian Technical Guide for the anthropometric nutritional assessment of adults [20].

#### 2.4.3. Body Fat Percentage (%BF)

%BF was measured through bioelectrical impedance analysis (InBody 520, Biospace Co. Ltd., Seoul, Korea). The measurement of the total and segmental body composition of the body (arms, legs, and trunk) using the InBody 520 system was made at 5.50 and 500 kHz. The InBody 520 was previously validated in 68 healthy women and 42 men aged 21 to 82 years [21]. The following criteria were used for their classification: low-fat, normal, high-fat, and very high-fat. These classification criteria are used in both men and women. The procedures were performed according to the manufacturer's instructions, which were based on the work of Gallagher et al. [22]. However, it is worth mentioning that there is a debate about which classification criteria to use [23]. All anthropometric evaluations were carried out in the facilities of the Nutrition Clinic of the Universidad Peruana Unión by a professional nutritionist.

### 2.5. Statistical Analysis

The statistical software package SPSS, version 25 (SPSS Inc., Chicago, IL, USA) was used for data processing and analysis. The descriptive analysis of the sociodemographic and educational information was carried out by using tables of absolute frequencies and percentages. The chi-square test was used to evaluate the difference in sociodemographic variables between male and female students. To determine the association between the various potential risk factors with cardiometabolic risk, a logistic regression analysis was used. This type of regression analyzed the relationship between the dependent variable, CMR, and some independent variables, controlling for possible confounding factors. The independent variables were chosen by theoretical relationship with the dependent variable and by means of a bivariate logistic regression model. All variables with a probability value (*p* value) lower than 0.05 in the bivariate analysis were included in the multivariate logistic regression analysis. The odds ratio (OR) and its 95% confidence interval (IC) were calculated to identify the factors associated with CMR.

## 3. Results

A total of 2048 university students were considered for this study, of which 56.7% (*n* = 1162) were men (Table 1). Most were single (98.1% (*n* = 2009)) and 43.3% (*n* = 886), women. The students of the Health Sciences careers of this study (Human Nutrition, Nursing, Psychology, and Medicine), represented the highest proportion of the sample (38.5% (*n* = 789)), followed by those of engineering (29.2% (*n* = 599)) (Table 1).

**Figure 2:**
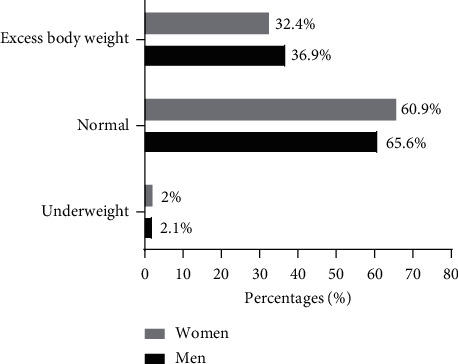
Distribution of the descriptive characteristics of the BMI according to the sex of the students. *Note*. *p*=0.097, chi-square was used to compare the proportions between women and men. Underweight (BMI <18.5 kg/m^2^), normal (BMI 18.5–24.9 kg/m^2^), and excess body weight (BMI ≥25).

**Figure 3:**
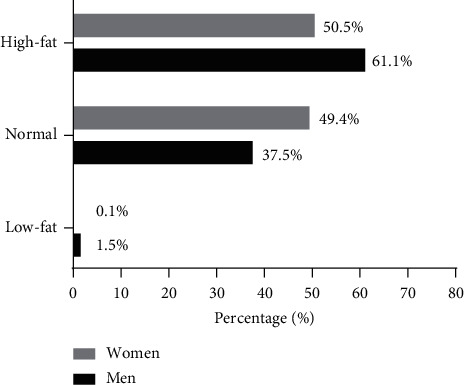
Distribution of the descriptive characteristics of %BF according to the sex of the students. *Note*. *p* < 0.001, chi-square was used to compare the proportions between women and men.

Figures 2 and 3 show the descriptive characteristics of the BMI and %BF of the university students according to their sex. The highest proportion of men was overweight compared to women (36.9% vs. 32.4%). The same is observed for %BF, where 61.1% of men had excess body fat (*p* < 0.001).

Our final multiple logistic regression model considered the CMR as a dependent variable. The students were categorized with CMR and no CMR as follows: WC ≥94 cm in men and ≥80 cm in women, with CMR and WC <94 cm in men and <80 cm in women, no CMR [20]. The independent variables such as sex, nationality, health sciences students, excess body weight, and %BF were considered as predictors of CMR because they were significantly related in the bivariate analysis. Consequently, women (OR, 0.22; 95% CI, 0.17, 0.29), Peruvian students (OR, 0.59; 95% CI, 0.39, 0.90), and health science students (OR = 0.76; 95% CI, 0.62, 0.94) appear to be less likely to manifest CMR. Moreover, excess body weight (OR, 17.28; 95% CI, 13.21, 22.59) and a high %BF (OR, 4.55; 95% CI, 3.55, 5.84) were strongly associated with the CMR (Table 2).

## 4. Discussion

In this study that analyzed excess body weight, %BF, and WC as a factor associated with CMR among Peruvian university students, several notable findings were evidenced. The highest proportion of men had excess body weight and a high %BF. Excess weight and body fat are strongly associated with CMR. However, women, Peruvian students, and health science students were less likely to have CMR.

Although some studies have shown that the probability of being overweight or obese is approximately the same between men and women [24], however, in the current study, men had higher excess body weight compared to women. These results coincide with the findings of a previous study carried out in university students, in which it was found that men had greater excess body weight [25]. Similarly, other studies showed statistically significant differences in terms of obesity between men and women [16, 26, 27]. Epidemiological evidence available in Western countries shows an increasing trend of obesity in males than females [28]. This trend could be due to the fact that women are more susceptible to social and family pressures to maintain an acceptable body image, which, consequently, can make them more sensitive and concerned about their weight status. Despite the prevalence of obesity in men and the existing evidence linking excess body weight to noncommunicable diseases, men appear to have little interest in participating in nutrition education programs to achieve healthy weight loss [29]. In fact, men generally care less about their appearance and body weight than women; in addition, they lack basic knowledge about healthy eating and nutrition [30]. The reduction of excess body weight in both men and women is an essential step in preventive medicine considering that it can guarantee optimal health in university students and a better quality of life in adulthood [31].

The identification of %BF and excess body weight are key anthropometric indicators that allow health professionals to detect patterns of unhealthy or risky lifestyles both in university students and in the general population [32]. In our study, men had a higher %BF than women. A potential mechanism for the differences in body composition between men and women is that, during puberty, men tend to accumulate more visceral fat than women [33]. This happens even in normal weight and non-obese men, which could lead to a higher CMR by accumulating more fat in the abdominal region [34]. Both the results in terms of obesity and fat percentage seem to be linked to the fact that women are more motivated to control their diet to maintain a healthy body [35]. Our findings disagreed with the report of a recent study conducted on Saudi college students, which showed that female students had a significantly higher mean body fat percentage compared to males [16]. Similarly, the results of the current study do not agree with the findings reported by Wilson et al. [25], who found that the value of the fat percentage was higher in women than in men.

Another important finding from this study is that women were less likely to have CMR. The incidence of cardiometabolic diseases differs between the sexes, and there appears to be a higher prevalence in men [36, 37]. A similar study found that women have lower CMR than men [38]. These results are consistent with findings from other studies showing that men have a higher risk of developing cardiometabolic diseases according to anthropometric indicators such as BMI, WC, and waist-hip ratio (WHR) [16, 36]. There are various mechanisms that could explain the lower CMR in women. For example, women have more fat accumulated in the lower extremities (pear-shaped body type) and less lean mass compared to men who tend to accumulate more abdominal fat (apple-shaped body type) [39]. The accumulation of fat in the lower body of women is a relatively protective factor against CMR [38, 39].

In this study, Peruvian students were less likely to present CMR compared to international students. It is possible that the higher CMR observed in international students is due to the negative effects of the adaptability of the new environment, which could also be explained by sociocultural issues. Furthermore, the differences between the lifestyles and eating habits between the two groups could justify these results. Furthermore, it is important to emphasize that international students are mostly susceptible to adopting new lifestyle choices [40], for example, healthy or unhealthy eating habits, adequate or inappropriate physical exercise routines, among others [41].

It is expected that students enrolled in faculties of health sciences have a better knowledge about the importance of choosing a healthy lifestyle and that this, in turn, reflects on health status, particularly on body composition [42]. In our study, being a health science student is a protective factor against CMRs. In line with our results, a similar study has reported an optimal health status for indicators such as BMI, blood pressure, and WC in university students of health sciences [43]. For students enrolled in faculties of health sciences, optimal health represents a favorable indicator, being future professionals and health models [44]. The current health status of health science students is a key predictor of future active participation in clinical counseling and noncommunicable disease prevention and health promotion activities. Considering that the population sees health professionals as a role model, students enrolled in health sciences faculties with suboptimal health status may pose a risk to the health and well-being of the population in the future. Health professionals leave a strong impression on their patients regarding to practicing healthy living recommendations [45].

The results of the current study indicate that excess body weight and a high percentage of fat are strongly associated with CMR. Both global obesity and a high percentage of fat are predictors of CMR, considering that the accumulation of visceral fat can lead to cardiometabolic diseases [46]. Therefore, in addition to global obesity, the measurement of adiposity parameters could help identify students at increased risk for cardiovascular disease [47]. A nutritional intervention and constant monitoring in college students would be helpful to address or reduce the risk of the aforementioned diseases and risk factors [48].

In the current study, for the prediction of cardiometabolic events, WC was considered as one of the best anthropometric indicators of clinical importance to predict morbidity and risk of death [3, 38]. WC is a simple but useful method to evaluate and reflect the distribution of fat in the abdominal area, which can be of importance in the prevention of cardiometabolic diseases such as cardiovascular diseases and type 2 diabetes [49, 50]. Faced with the increasing prevalence of abdominal obesity and the increased risk of cardiometabolic diseases, it is important to carry out adequate nutritional interventions in the population to reduce and maintain WC in an optimal state. The literature shows that regardless of age and sex, a decrease in energy intake through a healthy diet or an increase in energy expenditure through a physical exercise routine is associated with a significant reduction in waist circumference [3, 51]. Being a WC a marker of morbidity [50], therefore, it is suggested that it should be routinely measured in clinical practice and nutritional counseling to provide additional information to guide the proper management and approach of patients [3].

### 4.1. Strengths and Limitations

The notable strength of the current study was the availability of anthropometric data related to more than 2,000 college students. However, this strength must be considered in light of the limitations of the study. First, the study design was cross-sectional, and, therefore, its interpretation is limited to the temporality of cause and effect. Also, the fact that we have used a selective group of students from a private university is a limiting factor because they do not represent the general student population. Likewise, the anthropometric indicators were measured once, whereas two or three repetitions are normally needed in some cases [52]; in addition, the bioelectrical impedance analysis (BIA) method was used to determine the %BF, which is less accurate compared to DEXA; however, BIA is a reproducible method used in several epidemiological studies to evaluate %BF and other measurements of body composition [53]. Likewise, some factors that could influence BIA results such as menstrual cycle, physical activity, food, and beverage intake, were not considered in our study. In addition, hip circumference was not included; therefore waist-hip ratio was not measured. Finally, we have not considered other factors such as dietary intake and physical activity, which are other important determinants of body composition and that they are also considered as cardiometabolic risk factors. This is due to the scarcity of this information.

## 5. Conclusion

The findings of the present study highlight the prevalence of excess body weight and adiposity and CMR in male Peruvian students. Considering that cardiometabolic diseases and adiposity represent a higher risk of mortality, high social cost, and damage to public health, the development of preventive programs based on nutritional education helps to detect early cardiometabolic risks in university students and it can be helpful in preventing any future adverse complications. Therefore, future research on this topic is needed, which covers the eating aspect and physical activity in this vulnerable population group.

## Figures and Tables

**Table 1:** Number and percentages of students according to their sociodemographic characteristics.

Variable	Women		Men		Total		*X* ^2^	*p* value
	*N*	%	*n*	%	*n*	%		
Age (years)								
20–25	619	53.3	385	43.5	1004	49.0	19.384	^*∗*^<0.001
26–29	543	46.7	501	56.5	1044	51.0		
Nationality								
Peruvian	1060	91.2	811	91.5	1871	91.4	0.062	0.803
Foreign	102	8.8	75	8.5	177	8.6		
Civil status								
Single	1150	99.0	859	97.0	2009	98.1	10.923	^*∗*^0.001
Married	12	1.0	27	3.0	39	1.9		
Faculty of study								
Business	176	15.1	123	13.9	299		353.653	^*∗*^<0.001
Engineering	280	24.1	319	36.0	599	29.2		
Education	121	10.4	67	7.6	188	9.2		
Health sciences	585	50.3	204	23.0	789	38.5		
Theology	0	0.0	173	19.5	173	8.4		

^*∗*^ *p* < 0.05 (significant).

**Table 2:** Odds ratio for CMR in university students.

Variable	(95% CI)	*p* value
Sex		
Women	0.22 (0.17, 0.29)	<0.001
Men	1.00	
Nationality		
Peruvian	0.59 (0.39, 0.90)	0.014
Foreign	1.00	
Health science students		
Yes	0.76 (0.62, 0.94)	<0.001
No	1.00	
Excess body weight		
Yes	17.28 (13.21, 22.59)	<0.001
No	1.00	
Excess body fat		
Yes	4.55 (3.55, 5.84)	<0.001
No	1.00	

CMR, cardiometabolic risk; OR, odds ratio; CI, confidence interval; *p* < 0.05 (significant).

## Data Availability

The datasets used and analyzed during the present study are available from the corresponding author on reasonable request.
